# Special Issue “Role of Molecular Dynamics Simulations and Related Methods in Drug Discovery”

**DOI:** 10.3390/ijms27010264

**Published:** 2025-12-26

**Authors:** Huanxiang Liu

**Affiliations:** Faculty of Applied Sciences, Macao Polytechnic University, Macao SAR 999078, China; hxliu@mpu.edu.mo

Molecular dynamics (MD) simulations and related computational methodologies—such as binding free energy calculations and Markov state models—have become indispensable tools in modern drug discovery [1,2,3]. These approaches provide unique advantages for elucidating protein conformational dynamics, as well as for characterizing the thermodynamics and dissociation kinetics of protein–ligand interactions [4,5,6]. By revealing the molecular mechanisms underlying protein conformational changes, MD simulations offer critical insights into disease pathogenesis, receptor activation, and ion channel gating [7,8,9,10]. Such insights directly inform therapeutic strategies and facilitate the rational design of novel drugs. Through detailed analysis of interaction mechanisms, MD simulations deepen our understanding of how ligands modulate biological targets and provide essential guidance for lead compound optimization.

This Special Issue highlights the application of molecular dynamics simulations and allied computational techniques in advancing drug discovery. It features five original research articles that explore protein structure–function relationships, protein–ligand interactions, and ligand binding/unbinding dynamics.

The first study contribution 1 employed molecular docking, MD simulations, and free energy calculations to investigate the interaction mechanisms between the SARS-CoV-2 main protease (3CLpro) and three inhibitors: Bofutrelvir, Nirmatrelvir, and Selinexor. Comparative analysis based on docking scores, structural dynamics from MD trajectories, and MM-GBSA binding affinities revealed that Nirmatrelvir—the active component of Paxlovid—exhibits the strongest binding affinity, confirming its potency as a 3CLpro inhibitor. This work provides novel insights into the dynamic stabilizing interactions between these inhibitors and the protease target.

In the second paper contribution 2, the authors investigate the binding and unbinding mechanisms of representative inhibitors to Mycobacterium tuberculosis DNA gyrase subunit B (GyrB), aiming to inform the design of new agents against multidrug-resistant tuberculosis. Using classical MD, tau-random acceleration MD (τ-RAMD), and steered MD (SMD) simulations, they elucidated the binding and dissociation mechanisms of two representative inhibitors, novobiocin and SPR719. Classical MD simulations indicated that both electrostatic and van der Waals interactions contribute favorably to binding, with key residues including Asn52, Asp79, Arg82, Lys108, Tyr114, and Arg141. τ-RAMD simulations suggested that inhibitor dissociation occurs primarily through the ATP channel, while SMD simulations revealed that both inhibitors follow a similar dissociation pathway, requiring the disruption of hydrophobic and hydrogen-bonding interactions within the ATP active site. These findings offer valuable guidance for the structure-based design of novel GyrB inhibitors.

In the third contribution contribution 3, molecular dynamics simulations were employed to investigate the dissociation processes of antagonists from c-Src. The study is grounded in the perspective that, beyond binding affinity, the residence time (RT) of a drug–target complex critically influences drug efficacy and selectivity. As c-Src represents a key therapeutic target in multiple cancers, the development of antagonists with prolonged RT holds promise for improving treatment outcomes. Using Gaussian accelerated molecular dynamics (GaMD) simulations, the authors elucidated the structural basis of interactions between c-Src and selected antagonists, identifying key features associated with differing residence times. The results reveal that the long-RT compound DAS-DFGO-I (DFGO) occupies an allosteric site, forming hydrogen bonds with residues E310 and D404 and engaging in hydrophobic contacts with residues such as L322 and V377—interactions that collectively contribute to its extended RT. Further modification by replacing the amide group with a sulfonamide yielded a new derivative, DFOGS, which exhibited enhanced hydrogen-bond stability with E310 and D404, thereby increasing its binding stability to c-Src. These findings offer valuable theoretical insights for the rational design of long-RT c-Src inhibitors with improved selectivity and efficacy.

The fourth investigation contribution 4 focused on elucidating the protective mechanism of EGCG against Transthyretin Amyloidosis (ATTR). ATTR is a progressive systemic disorder driven by the misfolding and subsequent amyloid aggregation of transthyretin (TTR). Therapeutic strategies for this disease often aim to stabilize the native TTR tetramer and disrupt existing fibrillar aggregates. Building on prior evidence that epigallocatechin gallate (EGCG) may interfere with multiple stages of TTR aggregation, this study employed molecular dynamics simulations to dissect its interactions—both with the TTR tetramer and with amyloid-like aggregates formed by the pathogenic V30M variant. Simulations revealed that EGCG binds stably within the V30M TTR tetramer, forming hydrogen bonds with residues in the flexible AB-loop and EF-helix-loop regions, thereby markedly reducing their structural mobility. Furthermore, the polyaromatic nature of EGCG enhances local hydrophobicity at the binding interface, which impedes solvent accessibility and tetramer dissociation. Regarding preformed V30M-TTR aggregates, EGCG was observed to promote the dissociation of peripheral β-strands by disrupting key inter-residue interactions that maintain aggregate integrity. It can also intercalate between side chains of adjacent β-strands, effectively destabilizing the ordered amyloid architecture. Collectively, this work clarifies the dual mechanism by which EGCG mitigates TTR amyloidosis, offering a valuable theoretical foundation for future ATTR drug development.

In light of the significant role played by reactive oxygen species and lipid membrane interactions in the pathogenesis of several diseases, Meng et al. contribution 5 further investigated how membrane charge, electrolyte type, and glycosylation influence the distribution of the negatively charged superoxide anion radical (·O_2_^−^) near cellular membranes. Based on the constructed various phospholipid bilayer systems incorporating ·O_2_^−^, different electrolytes, and glycosylated components using Charmm-GUI and Amber16, they performed molecular dynamics simulations on these systems to evaluate ·O_2_^−^ behavior near the lipid interface under varying environmental conditions. The simulations revealed that, in contrast to sodium, potassium ions enhance the ability of negatively charged phospholipid membranes to locally deplete ·O_2_^−^ near the membrane surface. Moreover, glycosylation markedly decreased the density of ·O_2_^−^ adjacent to the phospholipid bilayer by 78.3% relative to neutral lipid membranes. This reduction suggests a potential mechanism by which glycosylation could suppress lipid peroxidation through decreased local ·O_2_^−^ concentration.

Collectively, these findings underscore the growing role of molecular dynamics simulations and related computational methods. By revealing the dynamic and energetic landscapes of drug–target interactions, they enable the rational design of inhibitors with optimized binding kinetics, shifting the focus from mere binding affinity toward sustained efficacy and enhanced selectivity. As computational power grows and algorithms become more sophisticated, the integration of these in silico techniques will continue to deepen, paving the way for more efficient and successful development of therapeutics against a wide range of diseases. The future of drug discovery is undeniably computational, dynamic, and driven by atomic-level insight.

## References

[1] Liu X., Shi D., Zhou S., Liu H., Liu H., Yao X. (2018). Molecular dynamics simulations and novel drug discovery. Expert Opin. Drug Discov..

[2] Lee M.R. (2025). From MM-PBSA to H-MMGB: Multiscale Modeling for Biomolecular Structure and Drug Discovery. J. Phys. Chem. B.

[3] Zhang Q., Gong X., Liu H., Yao X. (2025). Application of computational methods in the drug discovery and development of Alzheimer’s disease. Acta Pharm. Sin. B.

[4] Gong X., Tan S., Wang L., Yao X., Liu H. (2025). Well-Tempered Metadynamics Simulations Combined with Free Energy Landscape Analysis Uncover the Conformational Transition Pathway of the DYG Motif in LRRK2. ACS Chem. Neurosci..

[5] Xue W., Ban Y., Liu H., Yao X. (2014). Computational study on the drug resistance mechanism against HCV NS3/4A protease inhibitors vaniprevir and MK-5172 by the combination use of molecular dynamics simulation, residue interaction network, and substrate envelope analysis. J. Chem. Inf. Model..

[6] Zhang Q., Zhao N., Meng X., Yu F., Yao X., Liu H. (2022). The prediction of protein–ligand unbinding for modern drug discovery. Expert. Opin. Drug Discov..

[7] Strodel B. (2021). Amyloid aggregation simulations: Challenges, advances and perspectives. Curr. Opin. Struct. Biol..

[8] Miao Y., McCammon J.A. (2016). G-protein coupled receptors: Advances in simulation and drug discovery. Curr. Opin. Struct. Biol..

[9] Otun O., Aljamous C., Del Nero E., Arimont-Segura M., Bosma R., Zarzycka B., Girbau T., Leyrat C., de Graaf C., Leurs R. (2024). Conformational dynamics underlying atypical chemokine receptor 3 activation. Proc. Natl. Acad. Sci. USA.

[10] Wang L., Zhang Q., Tong H.H.Y., Yao X., Liu H., Li G. (2024). Computational methods for unlocking the secrets of potassium channels: Structure, mechanism, and drug design. Wiley Interdiscip. Rev. Comput. Mol. Sci..

